# Deep Ensemble Learning-Based Models for Diagnosis of COVID-19 from Chest CT Images

**DOI:** 10.3390/healthcare10010166

**Published:** 2022-01-15

**Authors:** Mohamed Mouhafid, Mokhtar Salah, Chi Yue, Kewen Xia

**Affiliations:** School of Electronics and Information Engineering, Hebei University of Technology, Tianjin 300401, China; engalmokhtar@gmail.com (M.S.); chiyueliuxin@126.com (C.Y.); kwxia@hebut.edu.cn (K.X.)

**Keywords:** coronavirus detection, deep learning, convolutional neural network, transfer learning, stacking, weighted average ensemble

## Abstract

Novel coronavirus (COVID-19) has been endangering human health and life since 2019. The timely quarantine, diagnosis, and treatment of infected people are the most necessary and important work. The most widely used method of detecting COVID-19 is real-time polymerase chain reaction (RT-PCR). Along with RT-PCR, computed tomography (CT) has become a vital technique in diagnosing and managing COVID-19 patients. COVID-19 reveals a number of radiological signatures that can be easily recognized through chest CT. These signatures must be analyzed by radiologists. It is, however, an error-prone and time-consuming process. Deep Learning-based methods can be used to perform automatic chest CT analysis, which may shorten the analysis time. The aim of this study is to design a robust and rapid medical recognition system to identify positive cases in chest CT images using three Ensemble Learning-based models. There are several techniques in Deep Learning for developing a detection system. In this paper, we employed Transfer Learning. With this technique, we can apply the knowledge obtained from a pre-trained Convolutional Neural Network (CNN) to a different but related task. In order to ensure the robustness of the proposed system for identifying positive cases in chest CT images, we used two Ensemble Learning methods namely Stacking and Weighted Average Ensemble (WAE) to combine the performances of three fine-tuned Base-Learners (VGG19, ResNet50, and DenseNet201). For Stacking, we explored 2-Levels and 3-Levels Stacking. The three generated Ensemble Learning-based models were trained on two chest CT datasets. A variety of common evaluation measures (accuracy, recall, precision, and F1-score) are used to perform a comparative analysis of each method. The experimental results show that the WAE method provides the most reliable performance, achieving a high recall value which is a desirable outcome in medical applications as it poses a greater risk if a true infected patient is not identified.

## 1. Introduction

Since December 2019, COVID-19 has been featured in the media as a severe health problem. This Severe Acute Respiratory Syndrome Coronavirus (SARS-CoV-2) is part of the coronavirus family that gets transmitted through direct contact or by fomites. Symptoms of coronavirus infection include fever, cough, fatigue, and a loss of taste. Coronavirus can cause severe respiratory problems such as pneumonia, lung disorders, and kidney malfunction in some cases. A serial interval of five to seven days and a reproduction rate of two to three people make the virus very dangerous [1]. Several people are healthy carriers of a virus, which causes between 5% and 10% of acute respiratory infections [2]. To stop the spread of the COVID-19 infection, the timely quarantine, diagnosis, and treatment of infected people are the most necessary and important work.

RT-PCR [3] and Enzyme-linked Immunosorbent Assay (ELISA) [4] are the most widely used methods for identifying the novel coronavirus. RT-PCR is the primary screening procedure for identifying COVID-19 cases as it can detect the virus’ RNA in lower respiratory tract samples. The samples are collected in various ways, including nasopharyngeal and oropharyngeal swabs. Most countries are experiencing a shortage of testing kits due to the rapid increase in the number of infected people. Therefore, it would be prudent to consider other methods of identifying COVID-19-contaminated patients so that they can be isolated and the impact of the pandemic on many people can be mitigated.

The use of Computed Tomography (CT) for the diagnosis of infected people is a complement to RT-PCR. As every hospital has CT imaging machines, COVID-19 detection based on CT imaging can be applied efficiently as a way to test infected patients, but it does require expert diagnosis and additional time. Therefore, Computer-aided Diagnosis (CAD) systems can be used to classify COVID-19 patients based on their chest CT images [5]. CT images can be employed for COVID-19 screening for the following reasons:

- Ability to detect the disease quickly and enable rapid diagnosis.
- Utilization of readily available and accessible radiological images.
- Utilization of these systems in isolation rooms, which eliminates the risk of transmission.

The use of Deep Learning-based techniques has made significant progress in recent years in terms of efficiency and prediction accuracy. They have proven their generalization ability in solving complex computer-vision problems, especially within the medical and biological fields, such as organs recognition [6], bacterial colony classification [7,8], and disease identification [9]. CNNs have demonstrated exceptional performance in the medical imaging field compared to other networks [10].

The following study presents an efficient Deep Learning-based CAD system for detecting COVID-19. We combined three well-known Deep Learning models (the Visual Geometry Group (VGG)-19 [11], the Residual Network (ResNet)-50 [12], and the Densely Connected Convolutional Network (DenseNet)-201 [13]) using Stacking and Weighted Average Ensemble (WAE), following the basic philosophy that the performance is better with a combination of various classifiers than with individual classifiers. Further, the insufficient training data issue was resolved by using Data Augmentation technique [14], which enhance the training dataset by adding the transformed original instances. The performance of the system we proposed makes it clear that CT images can be employed in a real-world scenario for the detection of COVID-19. The contributions of this paper are as follows:

- A set of Ensemble Learning-based models was proposed to detect COVID-19 infected patients, extending the standard by modifying the topology of three well-recognized CNNs and picking the optimal set of hyper-parameters for network training.
- The proposed Ensemble Learning-based models were tested using two different chest CT-scan datasets.
- Various strategies were used to deal with the small datasets, including fine-tuning, regularization, checkpoint callback, and data augmentation.
- For the first time, the concept of WAE is applied to the specific COVID-19 detection problem, achieving a high level of performance compared to the existing methods.

The paper is organized in the following manner. Section 2 discusses the related work. Section 3 describes the proposed three Ensemble Learning-based models for the detection of COVID-19 from chest CT images. Section 4 presents the experimental results. Section 5 provides discussions of the results. Finally, Section 6 includes the conclusion.

## 2. Related Work

Due to the evolution of medical image processing techniques, the development of intelligent diagnosis and prediction tools began to emerge at a rapid pace [15]. The use of Machine Learning methods is widely accepted as a useful tool for improving the diagnosis and prediction of many diseases [16,17]. Feature extraction techniques are, however, necessary to obtain better Machine Learning models. Therefore, Deep Learning models have been broadly accepted in medical imaging systems due to their ability of extracting features automatically or by using pre-trained models such as ResNet [18].

When COVID-19 first emerged, the main challenge was the lack of datasets for testing and building Deep Learning models [19,20]. A private dataset was used by Xu et al. [21] to demonstrate how chest X-rays and chest CT scans can be used to detect COVID-19. They collected a total of 618 CT samples, achieving an overall accuracy of 86.7%. Yang et al. [22] published a public dataset that included 349 COVID-19 (+) scans from 216 patients and 463 COVID-19 (−) scans from 55 patients. A prominent radiologist who has been treating and diagnosing infected patients since the beginning of this epidemic confirms the value of their dataset. Their diagnosis techniques relied on self-supervised learning and multi-task learning, and they reported an accuracy of 89% and an F1-score of 90%. Wang et al. [23] introduced an open-access benchmark dataset (COVID-x), consisting of 13,975 Chest X-ray (CXR) images across 13,870 patient cases from five open-access data repositories. Their model obtained an accuracy of 93% which was later enhanced by Farooq et al. [24], with an accuracy of 96%. He et al. [25] provided another publicly-available dataset comprising of 349 COVID-19-positive CT images. In order to avoid overfitting, they proposed a self-supervised Transfer Learning technique that learns unbiased and powerful feature representations. Their methods achieved an Area Under Curve (AUC) of 94% and an F1-score of 85%.

In the wake of the dissemination of public chest X-rays and CT scans, researchers focused their efforts on developing Deep Learning models with a low average classification time and high accuracy [26,27]. Loey et al. [28] presented Conditional Generative Adversarial Nets (CGAN) along with classic Data Augmentation techniques based on a deep Transfer Learning approach. The use of classical Data Augmentation and CGAN assisted in increasing the CT dataset and solving the overfitting issue. Moreover, they selected five deep Transfer Learning models (VGGNet16, VGGNet19, ResNet50, AlexNet, and GoogleNet) for investigation. Their experimental results demonstrated that ResNet50 outperformed the other four deep models in detecting COVID-19 from a chest CT dataset. Polsinelli et al. [29] presented a light CNN design based on the SqueezeNet architecture to discriminate between COVID-19 and other CT scans (community-acquired pneumonia and healthy images). Their proposed model outperformed the original SqueezeNet on both dataset arrangements, obtaining an accuracy of 83%, a precision of 81%, an F1-score of 83%, and a recall of 85%. Lokwani et al. [30] identified the site of infection using a two-dimensional segmentation model based on U-Net architecture. Their model was trained using full CT scans from a private Indian Hospital and a set of open-source images, available as individual CT slices. They reported a specificity of 0.88 (95% Confidence Interval: 0.82–0.94) and a sensitivity of 0.96 (95% Confidence Interval: 0.88–1).

Another challenge is extracting features from chest CT images for the detection of COVID-19 [31]. Wang et al. [32] presented a joint learning strategy for COVID-19 CT identification that learns efficiently with heterogeneous datasets from various data sources. They created a strong backbone by rebuilding the recently suggested COVID-Net from the architecture and learning approach. On top of their improved backbone, they performed separate feature normalization in latent space to reduce the cross-site data heterogeneity. Their method outperformed the original COVID-Net on two large-scale public datasets. A new hybrid feature selection method was proposed by Shaban et al. [33], which combined both wrapper and filter feature selection methods. Almost all of the models used Deep Learning to extract the features [34,35,36].

The researchers employed the Transfer Learning technique to reach high accuracy and low computation time in COVID-19 detection [37], and among VGG16, VGG19, ResNet50, GoogleNet, and AlexNet, ResNet50 achieved the highest level of accuracy. Taresh et al. [38] evaluated the ability of different state-of-the-art pre-trained CNNs in predicting COVID-19-positive cases accurately from chest X-ray scans. The dataset employed in their experiments includes 1200 CXR scans from COVID-19 patients, 1345 CXR scans from viral pneumonia patients, and 1341 CXR scans from healthy people. Their experimental findings demonstrated the superiority of VGG16, MobileNet, InceptionV3, and DenseNet169 in detecting COVID-19 CXR images with excellent accuracy and sensitivity. Rahimzadeh et al. [39] came up with a robust method for increasing the accuracy of CNNs by adopting the ResNet50V2 network with a modified feature selection pyramid network. They presented a new dataset of 48,260 CT scans from 282 healthy people and 15,589 images from 95 COVID-19 patients. Their technique was tested in two ways: one on over 7796 scans and the other on about 245 patients and 41,892 scans of varying thicknesses. They were capable of recognizing 234 of the 245 patients, achieving an accuracy of 98%. Azemin et al. [40] used a Deep Learning approach based on the ResNet101 model. They employed thousands of readily available chest radiograph scans for training, validation, and testing and achieved an accuracy of 71%, an AUC of 82%, a specificity of 71%, and a recall of 77%.

As can be observed, the majority of the recent studies on COVID19 detection have relied on individual Deep Learning models e.g., AlexNet, VGG16, VGG19, ResNet50, and ResNet101 [28,38,40]. None of the studies attempted to combine the models in order to increase their detection capabilities except for one investigation by Ebenezer et al. [41] which has proposed a stacked ensemble that includes four pre-trained CNN networks (VGG19, ResNet101, DenseNet169, and WideResNet50-2) to detect COVID-19. Their stacked ensemble system was generated using a similarity measure and a systematic approach. On three different chest CT datasets, their system reached high recall and accuracy, outperforming the baseline models.

Another point to note is that most of the mentioned literature employed a single dataset to evaluate the performance, which is not sufficient when dealing with a medical scenario such as this [21,22,23,24,25,28,33,34,35,38,39,40]. Table 1 summarizes the aforementioned state-of-the-art methods.

In this study, we analyzed and discussed the benefits of employing ensemble techniques. By exploring the differences in performance levels between Stacking and WAE, we demonstrated the superior performance provided by WAE. Additionally, valuable findings were obtained while modifying pre-trained VGG19, ResNet50, and DenseNet201 models and fine-tuning our own dense classifier. Moreover, we conducted experiments on two different chest CT-scan datasets and compared the performances of the individual models, ensemble models, and existing models using the most used evaluation metrics in Machine Learning.

We built on the usage of Transfer Learning and ensemble techniques to complete three major goals.

- Develop a medical recognition system by employing Transfer Learning approach on state-of-the-art CNN models and combining them to form an ensemble using two Ensemble Learning techniques that may be readily duplicated by Deep Learning practitioners and researchers who may benefit from the present work to combat COVID-19.
- Achieve competitive performance by attaining high levels of accuracy, precision, recall, and F1-score on both datasets.
- Present and elaborate on the limitations of dealing with small datasets in important and sensitive tasks such as diagnosing COVID-19, as well as how fine-tuning, regularization, checkpoint callback, and data augmentation techniques can be used to overcome them.

## 3. Materials and Methods

**Figure 1:**
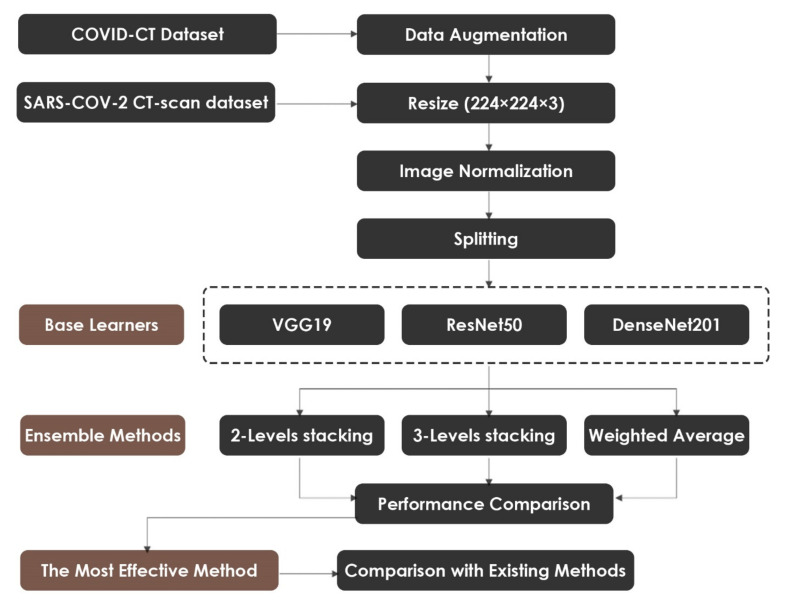
Flowchart of the Ensemble Learning framework.

In this section, we describe the proposed method for detecting COVID-19 using CT images. First, we explain the data preparation process, which includes Data Augmentation, Data Splitting, Image Resizing, and Image Normalization. Then, we present the process of fine-tuning the pre-trained VGG19, ResNet50, and DenseNet201 models. Lastly, we discuss the Ensemble Learning methods that were used to combine the modified networks. The overall workflow of the proposed methodology is depicted in Figure 1.

### 3.1. Data Preparation

**Figure 2:**
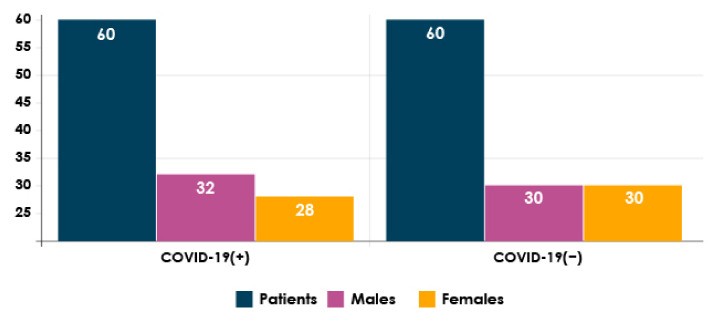
The detailed number of patients considered to compose SARS-CoV-2 CT-scan dataset [42].

**Figure 3:**
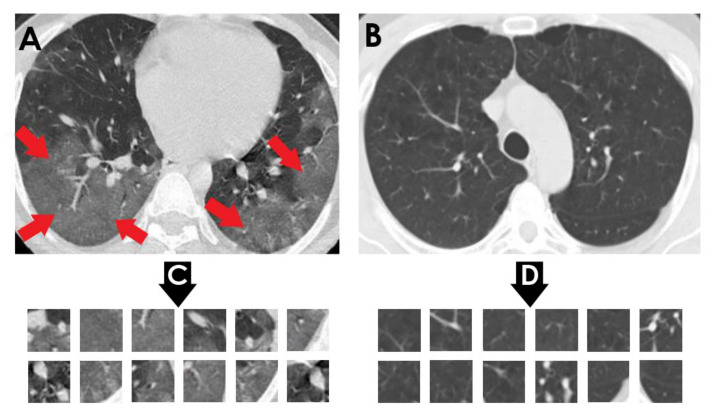
(**A**) Shows a CT of the lungs of COVID-19 (+) patient, in which a ground-glass opacity is visible in the lower lobes (red arrows). (**B**) Represents a CT of the lungs of COVID-19 (−) patient, in which there are no abnormalities. (**C**) Depicts infected patch samples. (**D**) Reflects non-infected patch samples. SARS-CoV-2 CT-scan dataset [42] is the source for these images.

**Figure 4:**
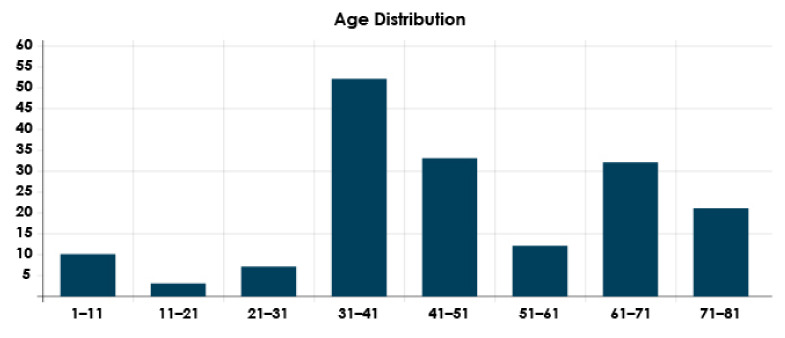
Age distribution of COVID-19 (+) patients.

**Figure 5:**
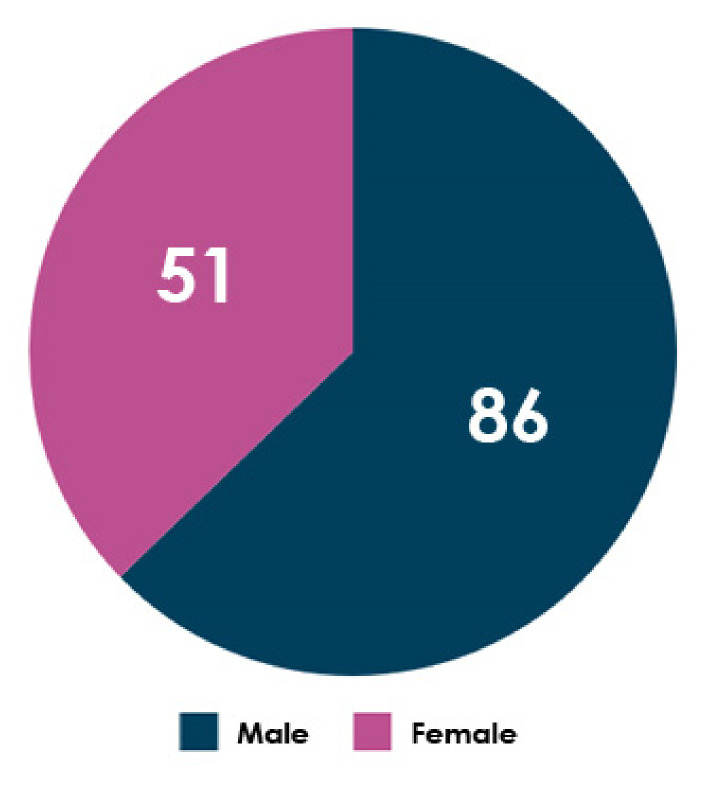
The gender ratio of COVID-19 (+) patients.

**Figure 6:**
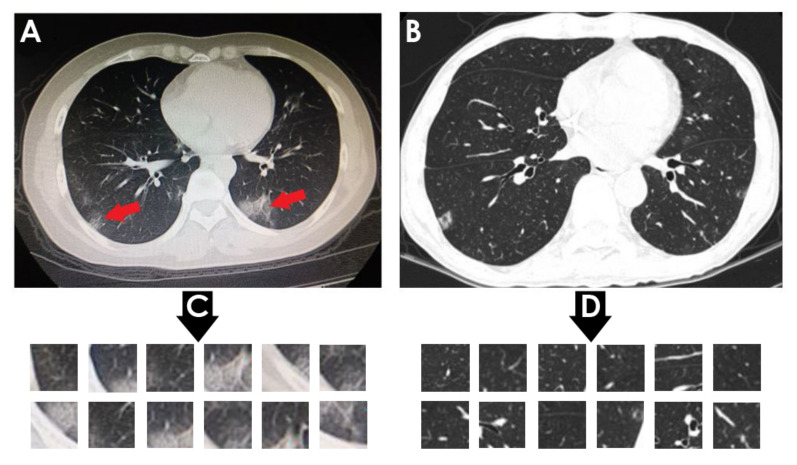
(**A**) Shows a CT of the lungs of COVID-19 (+) patient, in which there are multiple patchy ground-glass opacities in bilateral subpleural areas indicated by red arrows. (**B**) Represents a CT of the lungs of a COVID-19 (−) patient with normal controls. (**C**) Depicts infected patch samples. (**D**) Reflects non-infected patch samples. COVID-CT dataset [22] is the source for these images.

The proposed approach was tested using two chest CT scan datasets. The repositories from which our CT images were collected are as follows:

- SARS-CoV-2 CT-scan dataset by [42] from Kaggle (https://www.kaggle.com/plameneduardo/sarscov2-ctscan-dataset) (accessed on 2 December 2021): This dataset contains 2482 CT scan images, which are obtained from 120 patients and divided into 1252 COVID-19 (+) CT images and 1230 COVID-19 (−) CT images. The dataset was collected in 2020 from hospitals in Sao Paulo, Brazil. Figure 2 illustrates the detailed number of patients. The hospitals have not provided detailed characteristics of each patient due to ethical considerations. This dataset is constructed from digital scans of printed CT exams and has no standard image size (the dimensions of the largest images are 416 × 512 while the smallest images are 104 × 119). A comparison of COVID-19 (+) and COVID-19 (−) patients is shown in Figure 3. In Figure 3A, a ground-glass opacity is visible in the lower lobes. In Figure 3B, the chest CT scan shows no abnormalities. The patches that were sampled from infected areas and non-infected areas are shown in Figure 3C,D, respectively.
- COVID-CT dataset by [22] from GitHub (https://github.com/UCSD-AI4H/COVID-CT) (accessed on 2 December 2021): To assemble this dataset, COVID-19 (+) CT images were obtained from biRxiv and medRxiv repositories, posted from the 19 January 2020, to the 25 March 2020. The images were extracted using PyMuPDF software in order to maintain a high level of quality. The spatial sizes of the CT images range from 124 × 153 to 1485 × 1853. The meta data of each CT image (patient gender, age, medical history, scan time, location, severity of COVID-19, and radiology report) were manually collected. A total of 349 COVID-19 (+) CT images were obtained, from 216 patients. There are 169 patients whose age and 137 whose gender have been determined. The age distribution and the gender ratio of patients labeled with positive are shown in Figure 4 and Figure 5, respectively. It can be noted that the majority of COVID-19 patients are above the age of 30. In addition, the number of male patients is higher than the number of female patients, with 86 and 51, respectively. These patients are at varying stages of the disease on the 1st day through the 30th, with a majority as early as the 5th day and as late as the 10th day. The COVID-19 (−) CT images were collected from Radiopaedia website, from two other datasets (LUNA and MedPix), and from other articles and texts accessible through PubMed Central. A total of 463 COVID-19 (−) CT images were obtained from 55 patients. A comparison of COVID-19 (+) and COVID-19 (−) patients is shown in Figure 6. In Figure 6A, we can observe multiple patchy ground-glass opacities in bilateral subpleural areas. In Figure 6B, the chest CT scan shows the lungs with normal controls. In Figure 6C,D, we compare the patches from infected areas with those from non-infected areas, respectively. Our proposed system should subsequently be able to detect COVID-19 (+) patients by distinguishing between CT scans of patients infected with COVID-19 and those that are not.

Data Augmentation is applied to COVID-CT dataset [22] since it has fewer images than SARS-CoV-2 CT-scan dataset [42]. In Data Augmentation, multiple copies of the original image are produced with varying scales, orientations, locations, and brightness levels to enhance the volume of data and avoid overfitting [43]. Our image augmentation parameters were a rotation range of 10, width shift range of 0.1, height shift range of 0.1, shear range of 0.1, brightness range (from 0.3 to 1), and horizontal and vertical flipping. Besides Data Augmentation, we resized the CT images to (224 × 224 × 3) pixels since that is the size requirement of the three pre-trained CNN models employed in this work. Further, Image Normalization is used to establish a uniform data distribution by dividing the images by the number of channels, resulting in normalized data in the range of [0, 1]. This will ensure that the training of the deep models is more consistent. Data Splitting for training and validation is the last step. In both datasets, we used 80% for training and the remaining 20% for validation. Table 2 lists the CT scan images distribution for each dataset.

### 3.2. Transfer Learning

**Figure 7:**
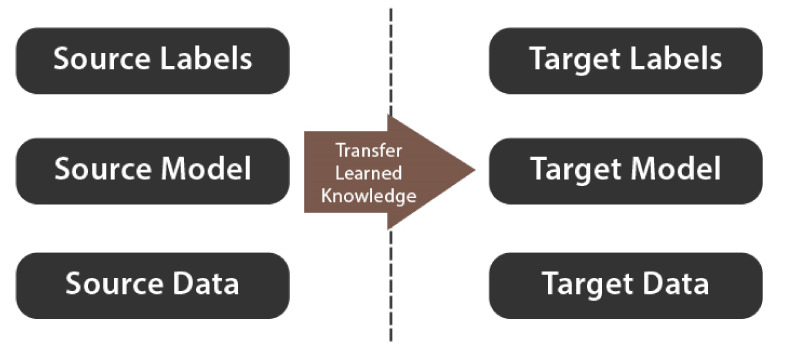
Transfer Learning approach.

Transfer Learning is the process of using the weights of a model that has been pre-trained on a different dataset to improve classification results on the current dataset. Figure 7 illustrates the basic concept of Transfer Learning. There are two types of Transfer Learning:

- **Feature Extraction:** This method uses a model that has been pre-trained on a standard dataset, such as ImageNet. The model’s classification part is then dropped. The remaining network is then used as a feature extractor, on which any classification algorithm can be performed [44].
- **Fine-tuning:** This method entails unfreezing the entire pre-trained model or part of it and retraining it on the new dataset [45].

For this study, pre-trained VGG19, ResNet50, and DenseNet201 are selected and fine-tuned according to our target datasets.

#### 3.2.1. Fine-Tuning of VGG19

VGG19 is a pre-trained network for classification. It consists of 19 layers (16 convolutional layers, 5 dense layers, 5 max-pooling layers, and a Softmax layer). From training on ImageNet, the parameters were used to solve a variety of problems such as classification of flowers [46], computer graphics [47], and fault diagnosis [48]. The network reached an accuracy of 90% with this dataset.

We highlight the following operations that compose VGG19: Convolution, Pooling, Flatten, Dense, Dropout, and Softmax.

**Figure 8:**
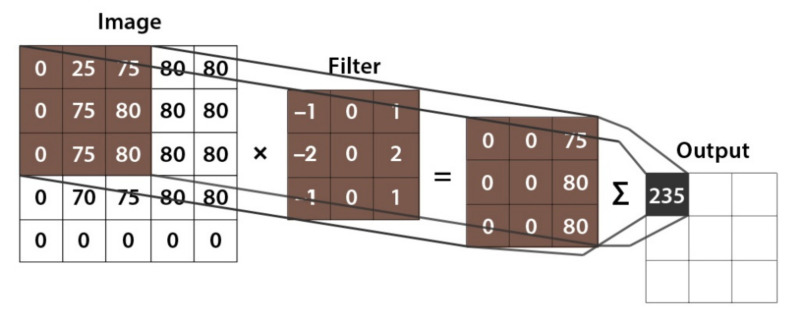
Representation of the convolution operation.

The convolutional layer is the main component of CNN. It performs what is known as a “convolution operation” which is a process that involves applying a filter to an input that produces an activation. Different features of an image can be extracted through convolutional layers, including textures, edges, objects, and scenes. The filter weights are updated during the training process, resulting in feature maps [49]. Figure 8 describes how the convolution operation works.

The pooling layer is used to reduce the dimension of the last layer and comes in two types: max-pooling and average-pooling. It can be regarded as a feature extractor when the convolution and pooling layers are combined [50].

**Figure 9:**
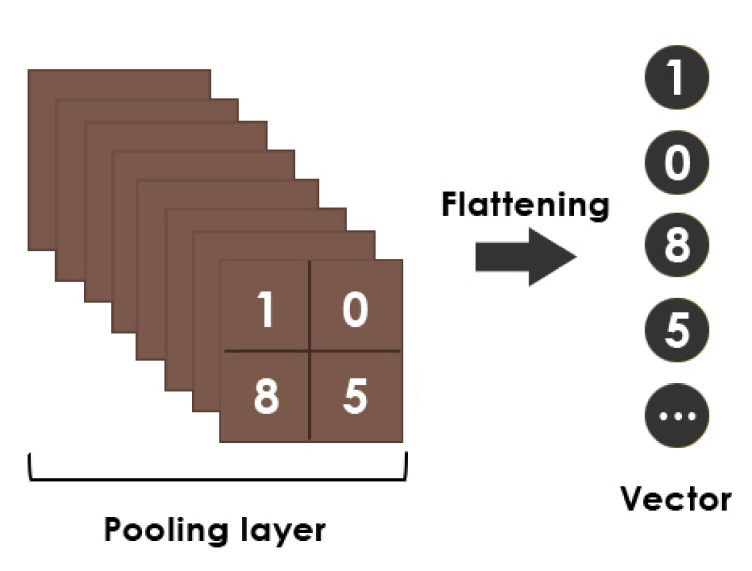
Representation of the flattening operation.

The flatten layer combines the output of the preceding layers into a single vector [51]. Figure 9 shows a simple flattening operation example.

The dense layer is used to link each neuron in a layer to each neuron in a previous or next one. Moreover, it can be considered as a classifier [52].

**Figure 10:**
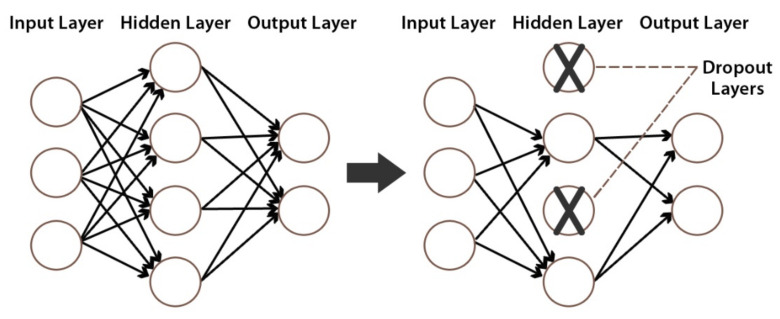
An example of a drop-out layer with a 50% drop-out probability.

Drop-out is a regularization operation that avoids overfitting by ignoring random neurons during training [53]. An example of a drop-out layer with a 50% drop-out probability is depicted in Figure 10.

Softmax is the most popular activation function employed in the output layer [54]. It calculates the probability score for each class. The mathematical representation of Softmax activation is shown in Equation (1). For i = 1, 2…, K and z = ($z_{1},\;z_{2}\ldots ,z_{K})\in \;{\mathbb{R}}^{K}$.

(1) $$\sigma {\left(z\right)}_{i}=\frac{e^{z_{i}}}{\sum_{j=1}^{K}e^{z_{j}}}$$

**Figure 11:**
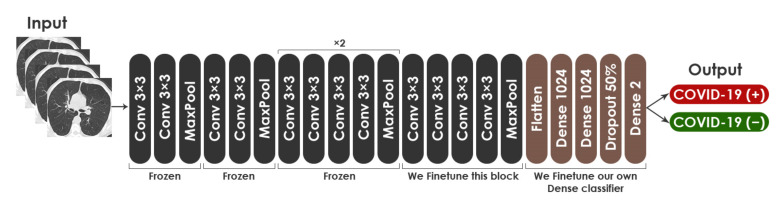
Architecture of modified VGG19. Conv: Convolutional Layer.

For fine-tuning the VGG19 network on CT images, we removed the top layers (dense layer and Softmax layer). Then, we used the last block for the training and froze the remaining four blocks. Lastly, we added new layers such as two dense layers, a drop-out layer, and a Softmax layer, at the top of the VGG19 network. The training hyper-parameters opted for this model are: (a) the cross-entropy loss function is used along with the Adaptive Moment Estimation (ADAM) optimizer [55], (b) mini-batch size is 32, (c) the training is performed up to 50 epochs, (d) the drop-out probability is 0.5, and (e) the specified learning rate for the training is 5 × 10^−5^. These hyper-parameters were found to be the best fit for network training through experiment. Figure 11 shows the proposed fine-tuned architecture based on VGG19 model. The architecture consisted of 23,174,210 total parameters, with 12,589,058 trainable parameters and 10,585,152 nontrainable parameters.

#### 3.2.2. Fine-Tuning of ResNet50

ResNet50 is a short name for Residual Network. The 50-layer network captures essential features and information about images that can be reused with smaller or similar dataset [23]. ResNet50 has other variants, including ResNet101, ResNet152, ResNet50V2, ResNet101V2, and ResNet152V2. For the classification of medical images, the use of ResNet has shown promising results [56]. ResNet50 was formed on the ImageNet dataset. In addition, it achieved an accuracy of 92.1%. The network comprises the identity and conv blocks. Moreover, 3 × 3 filters are used in the network’s convolutional layers and direct down sampling is achieved by the convolutional layers having a stride of 2. The final layer of the model is a dense layer with 256 and two channels, using ReLU and Softmax activation, respectively.

We describe the following concepts that compose ResNet50: ReLU activation function, stride, and identity function.

By using ReLU (Rectified Linear Unit) activation function, complicated functional mappings of inputs and response variables can be learned. In ReLU, a positive input will be directly generated, otherwise, it will result in zero. The mathematical formula of ReLU is shown in Equation (2).

(2) $$y=max\left(0,x\right)$$

Stride determines how the filter shifts around the input matrix. The mathematical formula for computing the output size for a convolutional layer is depicted in Equation (3). where o denotes the output height/length, k represents the filter size, w is said to be the input height/length, s is the stride, and p denotes the padding.

(3) $$o=\frac{\left(w-k+2p\right)}{s}+1$$

ResNet has overcome the issues associated with deep architectures by introducing a new neural network layer known as the Residual Block. Equation (4) illustrates the identity function, which is thought to be crucial in addressing the deep networks problem.

(4) $$F\left(x\right)=x$$

It is anticipated that by delivering the first layer’s input of the architecture as the last layer’s output, the model would continue to predict and learn whatever it had learned before the addition of input. The concepts of identity mapping and skip connection are defined by Equations (4) and (5). Identity mapping is a basic notion with no parameters. The addition of the output from descending layers to the previous layers is its main function.

(5) $$F\left(x\right)+x=H\left(x\right)$$

**Figure 12:**
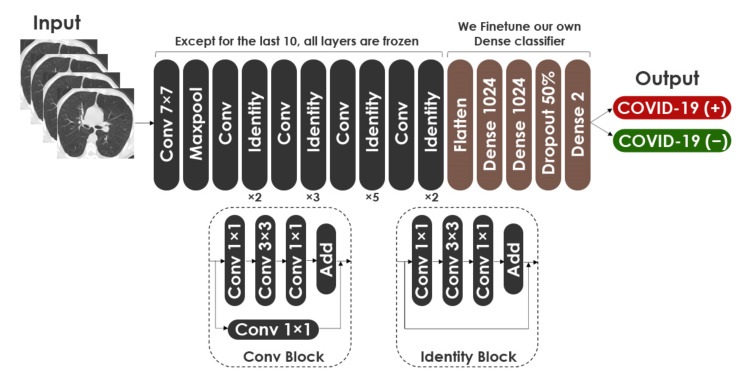
Architecture of modified ResNet50.

For fine-tuning the ResNet50 network on CT images, we removed the top layers. Then, we used the last ten layers for the training, and we froze the remaining layers. In the same way as VGG19, we added two dense layers, a drop-out layer and a Softmax layer, at the top of the network. The hyper-parameters for fine-tuning ResNet50 are the same as VGG19. Figure 12 describes the proposed fine-tuned architecture based on ResNet50 model. The architecture consisted of 43,514,754 total parameters, with 24,392,706 trainable parameters and 19,122,048 nontrainable parameters.

#### 3.2.3. Fine-Tuning of DenseNet201

DenseNet201 was developed by Huang et al. [13] in 2017. The network has demonstrated extraordinary performance on datasets such as CIFAR-100 [57] and ImageNet [58]. Trained on the ImageNet database, the model reaches 93.6% accuracy. Using DenseNet, the vanishing gradient problem can be alleviated, the propagation of feature maps can be enhanced, and parameters can be reduced. As compared to VGG [11] and ResNet [59], DenseNet has dense connectivity. The 201-layer network has other variants, including DenseNet121 and DenseNet169.

We explain the following notions that compose DenseNet201: Dense Block and Transition Layer.

DenseNet is made up of various Transition Blocks and Dense Blocks that overlap to construct a multilayer neural network. The internal Dense Block structure of the network employs the shortcut connection structure of the residual neural network. The residual neural network is typically made up of numerous residual block structures that overlay one other. A residual block is formed by connecting neighboring convolutional layers through a shortcut. The mathematical formula of the residual block mapping is represented in Equation (6). Where $H_{i+1}$ denotes the output, $H_{i}$ means the input, F is the identity mapping, and $W_{i}$ represents the weight.

(6) $$H_{i+1}=Re\;lu\left(H_{i}+F\left(H_{i,}W_{i}\right)\right)$$

Transition Layer primarily links two Dense Blocks. Each Transition Block has a convolution layer and average pooling layer to minimize the feature map size.

**Figure 13:**
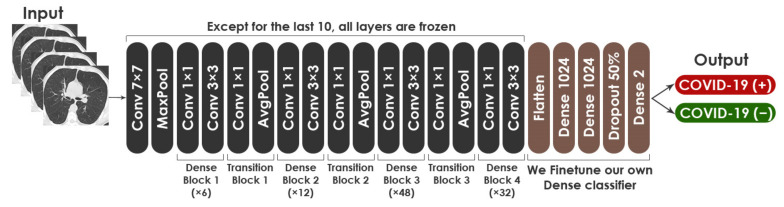
Architecture of modified DenseNet201.

For fine-tuning DenseNet-201, the top layers were removed, the last ten layers were kept trainable, and all other layers were untrainable. At the top of the network, we added two dense layers, a drop-out layer, and a Softmax layer, as we did with VGG19 and ResNet50. Similarly, the hyper-parameters for fine-tuning this network are the same as those for the aforementioned models. Figure 13 displays the proposed fine-tuned architecture based on DenseNet201 model. The architecture consisted of 27,238,978 total parameters, with 9,203,394 trainable parameters and 18,035,584 nontrainable parameters.

### 3.3. Ensemble Learning

Ensemble Learning is described in Machine Learning as the training of many models, called Base-Learners, and the combining of their prediction outputs to produce greater performance. The core idea is that by appropriately combining Base-Learners, robust models with higher accuracy can be created. Therefore, base models are employed in Ensemble Learning to construct generalized strong and more complicated models. In this study, we presented three Ensemble Learning-based systems, which are discussed in further detail in the following subsections.

#### 3.3.1. 2-Levels Stacking

**Figure 14:**
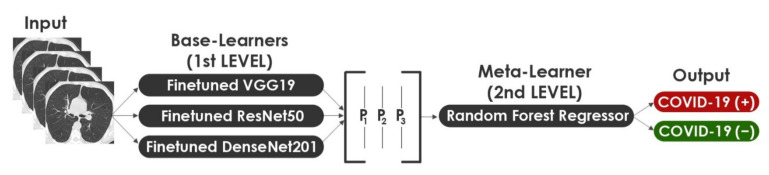
Representation of the 2-Levels Stacking approach.

In Machine Learning, Stacking is the process of combining more than one model to produce the best result. In order to reduce the errors in COVID-19 detection, we propose a 2-Levels Stacking approach by combining the outputs of three fine-tuned models. In this approach, we extend the standard 2-Levels Stacking method by choosing three strong modified models as Base-Learners. Our approach consists of two levels: level 1 is about training Base-Learners, while level 2 involves training a Meta-Learner. Each of the selected Base-Learners is trained separately. They are often complementary in that if one fails, the other succeeds. Taking advantage of this heterogeneity will allow the ensemble model to be constructed to improve the performance by combining all possible outputs. In level 1 learning, the stack of Base-Learners was trained concurrently on the original data and then the results were combined to give the new data for level 2. In level 2, the Meta-Learner takes as inputs the outputs $\left(p_{1},p_{2},{\;p}_{3}\right)$ of our three Base-Learners and learns to return final predictions. The fine-tuned VGG19, ResNet50, and DenseNet201 are the Base-Learners for level 1 while the Random Forest Regressor represents the Meta-Learner model for level 2. Figure 14 illustrates the 2-Levels Stacking approach.

Random Forest is made up of a number of classifiers, each of which contributes one vote to the assignment of the most repeated class to the input vector. The mathematical formula of Random Forest is introduced in Equation (7). Where $C_{b}\left(x\right)$ means the bth random forest tree’s class prediction.

(7) $$C_{rf}^{B}=majorityvote\;{\left\{C_{b}\left(x\right)\right\}}_{1}^{B}$$

In this paper, we used a Random Forest Regressor. The regression task here involves predicting the output probability of our two classes (COVID-19 (+) and COVID-19 (−)) depending on the output probability of the three fine-tuned Base-Learners. The classifier probabilities would also definitely work here using the Random Forest Classifier, but we opted to apply it for the 3-Levels Stacking method described in the following sub-section. The parameters selected for Random Forest Regressor are: (a) the number of trees in the forest (n_estimators) is 200, (b) the maximum depth of the tree (max_depth) is 15, (c) the number of jobs to run in parallel (n_jobs) is 20, and (d) the bare minimum of samples necessary for splitting an internal node (min_samples_split) is 20.

#### 3.3.2. 3-Levels Stacking

**Figure 15:**
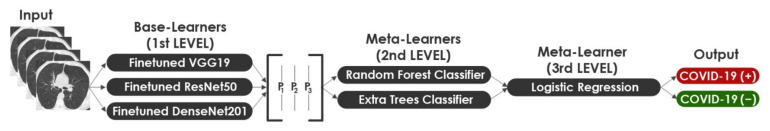
Representation of the 3-Levels Stacking approach.

3-Levels Stacking is an extension of Stacking, which involves Stacking with three layers. In level 1, we fit the same three Base-Learners that were used in the first approach. In level 2, instead of fitting a single Meta-Learner on the Base-Learners’ predictions, we fit two Meta-Learners. In level 3, we fit a last Meta-Learner that takes as inputs the predictions returned by the two Meta-Learners of the previous level. Figure 15 shows the architecture of our 3-Levels Stacking proposed mechanism. We limit the number of layers to three based on our observation that if the layer count was increased, we did not achieve significant improvements in model performance. The Random Forest Classifier and the Extra Trees Classifier represent the two chosen strong Meta-Learners for level 2 while Logistic Regression represents the selected Meta-Learner for level 3.

We highlight the following concepts that compose our 3-Levels Stacking approach: Extra Trees Classifier and Logistic Regression.

As an Ensemble Learning method, Extra Trees Classifier aggregates the outputs of various decorrelated decision trees obtained in a “forest” in order to produce its classification result. In principle, it is very equivalent to a Random Forest Classifier and differentiates only in the way the Decision Trees in the forest are constructed. The decorrelation of trees results from the random selection of trees. As a measure of the purity of node in Extra Tree Classifier, the Gini Index is used. It can be represented as shown in Equation (8) for a given dataset T. Where $\left(\frac{f\left(C_{i},T\right)}{\left|T\right|}\right)$ denotes the probability that a given case belongs to class $C_{i}$.

(8) $$\sum \sum_{j\neq i}\left(\frac{f\left(C_{i},T\right)}{\left|T\right|}\right)\left(\frac{f\left(C_{j},T\right)}{\left|T\right|}\right)$$

**Figure 16:**
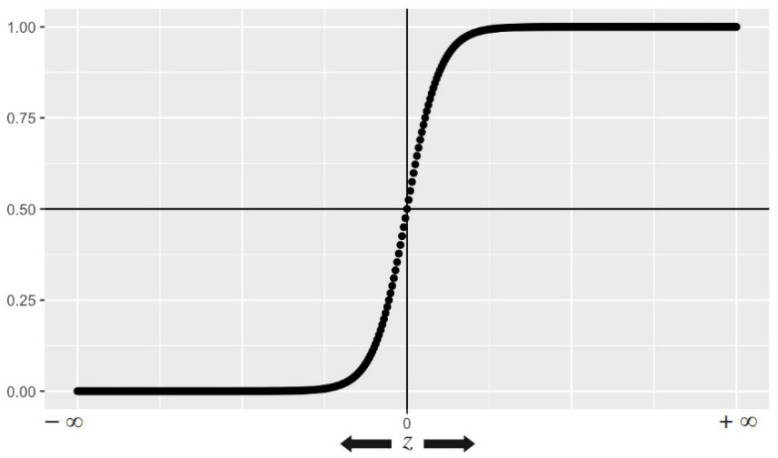
Representation of the Logistic Function (The values of this function have been plotted as z varies from −∞ to +∞).

Based on Logistic Regression, we can predict an outcome’s probability that only has two possible values. It generates a logistic curve with values ranging from 0 to 1. Figure 16 shows an illustration of the Logistic Function $f\left(z\right)$ (also known as inverse logit function or sigmoid function). Equation (9) depicts the mathematical formula on which the Logistic Regression model is based.

In our case, Logistic Regression takes as inputs the predictions returned by Random Forest Classifier and the Extra Trees Classifier.

The parameters selected for Random Forest Classifier and Extra Trees Classifier are listed in Table 3.

(9) $$f\left(z\right)=\frac{1}{1+e^{-z}}.\;$$

#### 3.3.3. WAE

**Figure 17:**
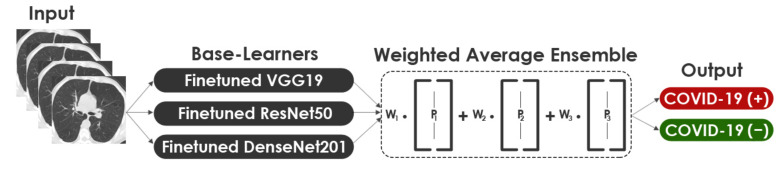
Representation of the Weighted Average Ensemble approach.

Model averaging is an Ensemble Learning strategy that involves all Base-Learners contributing a similar amount to the final prediction. In a Weighted Ensemble, the contribution of each Base-Learner to the last prediction is weighted according to its performance. A higher weight is given to Base-Learners that contribute more. In this approach, the calculated class probabilities for each Base-Learner $\left(p_{1},p_{2},\;p_{3}\right)$ were multiplied with the corresponding weights $\left(w_{1},w_{2},\;w_{3}\right)$ and the average obtained. In order to compare this approach with the 2-Levels and 3-Levels Stacking, we fitted the same three Base-Learners. An illustration of the structure of the WAE approach is shown in Figure 17. The mathematical formula of WAE is expressed in Equation (10). Where $w_{i}$ denotes the weight applied on the output of ith model which can be determined based on the model performance as shown in Equation (11). Where $DC_{i}$ represents $i{\mathrm{th}}^{}$ single model’s performance effectiveness.

(10) $$P\left(t\right)=\overset{N}{\underset{i=1}{\sum }}w_{i}p_{i}\left(t\right)$$

(11) $$w_{i}=\frac{DC_{i}}{\sum_{i=1}^{N}DC_{i}}$$

## 4. Results

This section investigates the performance of the proposed ensemble methods on two different datasets of chest CT scans: the SARS-CoV-2 CT-scan dataset [42] and the COVID-CT dataset [22].

### 4.1. Experiment Setup

All models of this study were implemented using the TensorFlow [60] library along with Keras [61]. DeepStack [62] was adopted to build the three Ensemble Learning-based models. All experiments were run using Google Colaboratory platform [63] with a virtual GPU powered by NVIDIA Tesla K80 and 12 GB RAM.

### 4.2. Performance Metrics

In this paper, four different metrics (accuracy, *precision*, *recall*, and *F1-score*) were used to evaluate the performances of the compared methods for COVID-19 detection. These are amongst the most used metrics in Machine Learning [64,65,66]. The following are the mathematical definitions for the evaluation metrics (in Equations (12)–(15), respectively):

(12) $$Accuracy=\frac{TruePositives+TrueNegatives}{TruePositives+FalsePositives+FalseNegatives+TrueNegatives}$$

(13) $$Precision=\frac{TruePositives}{TruePositives+FalsePositives}$$

(14) $$Recall=\frac{TruePositives}{TruePositives+FalseNegatives}$$

(15) $$F1-score=2\times \frac{Precision\times Recall}{Precision+Recall}$$

We define TruePositives, FalsePositives, TrueNegatives, and FalseNegatives as follows:

- TruePositives informs the number of COVID-19 (+) images predicted correctly as COVID-19 (+).
- FalsePositives informs the number of COVID-19 (−) images incorrectly predicted as COVID-19 (+).
- TrueNegatives informs the number of COVID-19 (−) images predicted correctly as COVID-19 (−).
- FalseNegatives informs the number of COVID-19 (+) images incorrectly predicted as COVID-19 (−).

### 4.3. Performance Analysis of the Base-Learners and the Ensemble Methods

In order to ensure comparable results, we trained all Base-Learners over 50 epochs using the same configuration. Keras checkpoint callback was executed at the end of each epoch to save when the validation accuracy improves. The hyper-parameters used for all Base-Learners are listed in Table 4. The runtime is a critical parameter for enhancing the efficiency of the proposed models. Table 5 and Table 6 compare the required time during the training process of the Base-Learners for the SARS-CoV-2 CT-scan dataset [42] and COVID-CT dataset [22], respectively. It is clear that the runtime varies from one model to another, which is primarily due to the total number of parameters for each model. The runtime became longer when the number of model parameters increased. With regard to COVID-CT dataset [22], the runtime and the time required for each epoch for the Base-Learners are longer than with respect to SARS-CoV-2 CT-scan dataset [42]. This is due to the use of Data Augmentation for COVID-CT dataset [22], which, as previously stated, has a low number of images. Another observation is that VGG19 had the shortest runtime and required the fewest epochs to converge on both datasets when compared to ResNet50 and DenseNet201. This is because VGG19 has fewer parameters (23,174,210) than the other models.

Table 7 and Table 8 show the performance evaluation metrics for the Base-Learners and Ensemble methods on the SARS-CoV-2 CT-scan dataset [42] and the COVID-CT dataset [22], respectively. For classification problems, accuracy is the primary measure. It refers to the degree of closeness between an estimated value and its original value in the classification process. Based on accuracy results, we observed a permutation in the rank order within the Base-Learners. However, this metric supported the superiority of all Ensemble methods compared to the average accuracy of the Base-Learners. As a result of using Ensemble Learning methods (2-Levels Stacking, 3-Levels Stacking, and WAE), the average accuracy of the Base-Learners has increased by 2.29%, 3.29%, and 3.29%, respectively, on the SARS-CoV-2 CT-scan dataset [42]. Regarding COVID-CT dataset [22], we noticed a remarkable increase in the average accuracy of the Base-Learners by 5.64%, 5.73%, and 6.73%, respectively. On both datasets, it was clear that the WAE method effectively improved the accuracy of the Base-Learners when compared to the other two Stacking methods. Furthermore, both Stacking methods produced the same level of accuracy on the SARS-CoV-2 CT-scan dataset [42], whereas the 3-Levels Stacking method yielded a slight accuracy increase of 0.09% over the 2-Levels Stacking method on the COVID-CT dataset [22].

**Figure 18:**
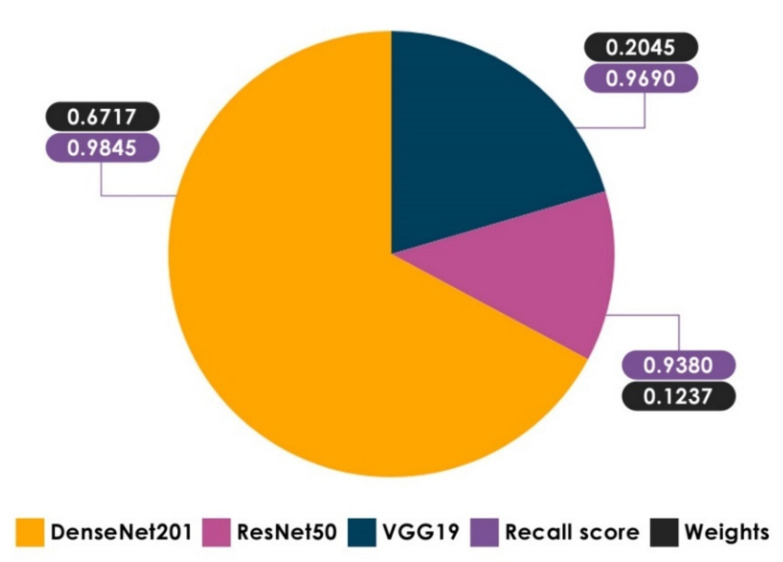
The optimal weights received for the Base-Learners based on the performance of the recall score function on the SARS-CoV-2 CT-scan dataset [42] (note that the weights range between 0 and 1).

Recall allows us to comprehend and measure the ability of the model to accurately recognize COVID-19 (+) patients. This metric is critical because false negatives can lead to the patients being misclassified as COVID-19 (−) when they are actually COVID-19 (+). Based on recall results, it is observed that the recall score of DzenseNet201 on the SARS-CoV-2 CT-scan dataset [42] is better than the other Base-Learners, highlighting the importance of combining the three Base-Learners, particularly using the WAE method, which gives a larger weight to Base-Learners that contribute the most, taking into account the results of each metric separately. Using DeepStack [64], weight optimization was carried out with a greedy randomized search relying on the Dirichlet distributions on the validation dataset. Figure 18 clearly shows that DenseNet201 has a higher weight when it comes to the recall score. Returning to the recall results of our proposed methods, the WAE method achieved the highest level of recall on both datasets with 99.22% and 95.28%, respectively, reducing the occurrences of false negatives. Considering the recall score of Stacking methods, it is clear that the average recall of the Base-Learners has increased by a significant margin on both datasets. It is encouraging that all Ensemble methods provide a recall of greater than 94.0%, indicating a low number of COVID-19 (+) patients incorrectly predicted as COVID-19 (−).

Precision is defined as the Positive Predictive Rate (PPR) and it is useful in limiting the spread of COVID-19 infection. Based on precision results, it can be noted that VGG19 outperformed the other Base-Learners on both datasets. A further observation is that WAE and 3-Levels Stacking methods produced the same level of precision on the SARS-CoV-2 CT-scan dataset [42], whereas the WAE method attained a considerable precision increase of 1.91% over the 3-Levels Stacking method on the COVID-CT dataset [22]. Regarding the Stacking methods, the 3-Levels Stacking achieved higher precision than the 2-Levels Stacking, demonstrating the significance of Stacking with more than two layers. Overall, all Ensemble methods provided over 93.0% precision, which means a lower burden on radiologists.

F1-score represents how well the classification has done in terms of recall and precision. Based on F1-score results, it is observed that the Ensemble methods achieved significantly more F1-score as compared with the average F1-score of the Base-Learners. The best F1-score was obtained using the WAE method, which achieved 98.65% and 94.93% on both datasets, respectively. 3-Levels Stacking was found to be the second-best Ensemble method. These high F1-scores indicate that we have a low number of false positives and false negatives. In this case, the model identifies the COVID-19 (+) patients and is not disturbed by the COVID-19 (+) cases incorrectly predicted as COVID-19 (−).

Our study results now provide evidence to prove the excellent findings obtained by the proposed Ensemble methods. By analyzing the performance metrics for each model, we can clearly see that the WAE method outperforms the modified CNN models and both Stacking models. As a result, it is deemed the chosen method to be compared with the existing methods. It is worth noting that the key difference is that the proposed WAE method assigns weights to each Base-Learner according to their own efficiency. In spite of the fact that the 2-Levels Stacking method failed to outperform fine-tuned VGG19 in terms of precision and F1-score on the SARS-CoV-2 CT-scan dataset [42] and across all metrics on the COVID-CT dataset [22], it showed a marked improvement after including the third level.

### 4.4. Comparison with State-of-the-Art Methods

We compared the performance of the proposed WAE method to the existing methods [25,28,29,32,33,41] on the respective datasets that were used to evaluate the existing methods. Our choice of these methods [25,28,29,32,33,41] for comparison was based on the dataset composition and the similarity of the experiments conducted. Accuracy, recall, precision, and F1-score were the evaluation metrics considered for the comparison. Table 9 and Table 10 compare the proposed method to existing methods on the datasets SARS-CoV-2 CT-scan dataset [42] and COVID-CT [22], respectively. It can be noted from Table 9 that the proposed method performed well on all four metrics compared to the existing methods. Table 10 show that the proposed method outperformed the existing methods in terms of recall, precision, and F1-score, making it the most efficient method for this COVID-19 binary-classification task.

### 4.5. Grad-CAM Visualizations

**Figure 19:**
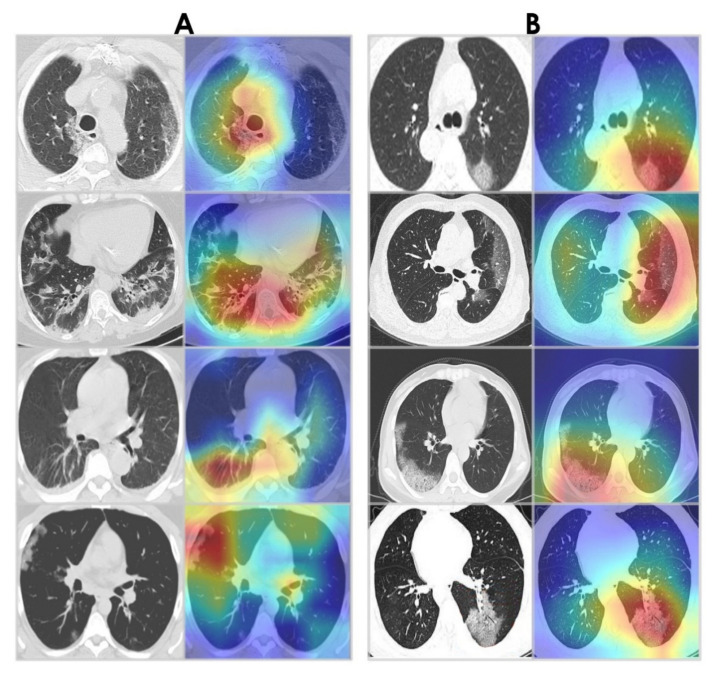
Grad-CAM visualizations. (**A**) Sample CT images from the SARS-CoV-2 CT-scan dataset [42]. (**B**) Sample CT images from the COVID-CT dataset [22].

Grad-CAM algorithm [67] was used to examine the behavior of the WAE network by visualizing the areas of infection in our chest CT images. This provides insight into what the network has learned and what part of its input contributed to detecting COVID-19. Grad-CAM visualizations for WAE are shown in Figure 19. Note that the WAE’s area of interest at the time of prediction is represented by the red and green visuals. It is noticed that the activations maps are focused on the lungs. Interestingly, in the majority of cases, WAE was able to localize the disease region based on relevant features from the chest CT images for both datasets.

## 5. Discussion

In this paper, we investigated two Ensemble Learning methods (Stacking and WAE) for detecting Covid-19 positive cases in chest CT images. We experimented Stacking with two and three levels. Each Ensemble Learning-based model was derived from a fusion of three fine-tuned CNNs: VGG19, ResNet50, and Densenet201. Two chest datasets were used to train and validate these networks. The Random Forest regressor algorithm was employed at the Meta-Learner level for 2-Levels Stacking to generate a final model. We picked Random Forest and Extra Trees classifiers as Meta-Learners for the second level of 3-Levels Stacking, and Logistic Regression for the third level. For all three methods, the same Base-Learners were used. The main difference between Stacking and WAE is that Stacking learns to combine the Base-Learners using a Meta-Learner. The WAE approach, on the other hand, does not include a Meta-Learner. The goal is to optimize the weights that are utilized for weighting the outputs of all Base-Learners and calculate the Weighted Average.

The small size of the datasets available was one of the major limitations of the current study. Despite this limitation, our proposed Ensemble Learning-based models were able to weed out false positives and false negatives and detect true positives and true negatives with a high level of performance on both datasets by employing strategies such as fine-tuning, drop-out, checkpoint callback, and data augmentation. To the best of our knowledge, this is the first paper to use WAE to detect COVID-19 from Chest CT scans. This method was found to be the most effective in this experiment, with > 98.5% accuracy on the SARS-CoV-2 CT-scan dataset [42] and >95% accuracy on the COVID-CT dataset [22]. These values are regarded as “extremely good” in the field of medical diagnosis and can be improved with a larger data set.

**Figure 20:**
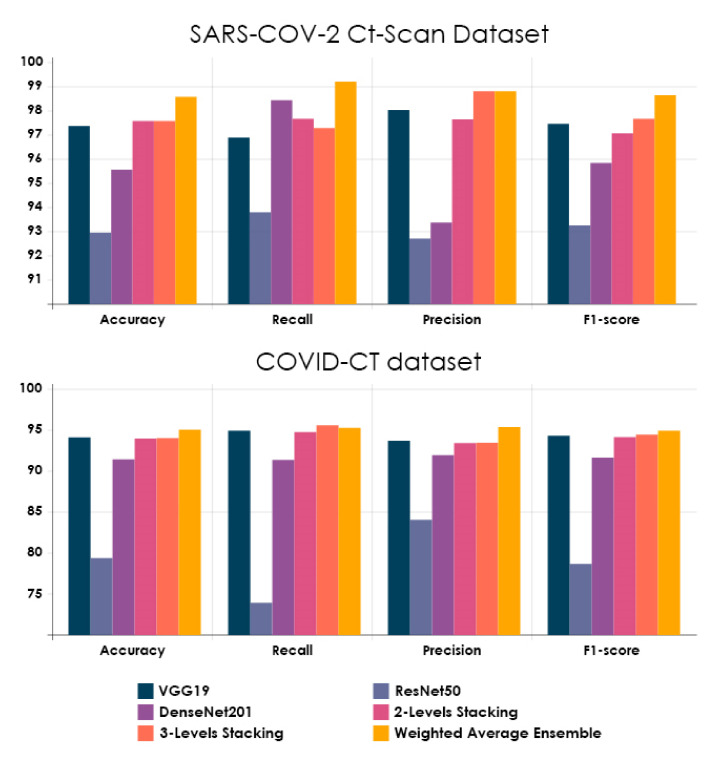
The performance evaluation metrics on both chest CT datasets for all studied models.

Fine-tuning was adopted on all three pre-trained CNN architectures using chest CT scans to enable networks to converge quickly and obtain features that are relevant to our study’s domain. It aided in the enhancement of the performance of these networks. VGG19, in particular, achieved a high level of performance on both datasets. Figure 20 summarizes all experimental results described in this paper.

The Ensemble Learning strategies used in this work have the considerable advantage of automating the randomization process, allowing the researcher to investigate multiple databases and capture useful insights. Rather than being restricted to a single classifier, they create many classifiers iteratively while randomly varying the inputs. By combining several single classifiers into one, we can obtain a more adaptive prediction scheme. In addition, these strategies can tackle the topic of RT-PCR kit lack of supply by requiring only a CT scan machine, which is already present in the majority of hospitals around the world. As a result, countries will no longer be forced to wait for RT-PCR kits’ large shipments.

The missing part of this work is that the models are yet to be validated during real clinical routines, so we are still in theoretical research mode. Therefore, we intend to evaluate our proposed models in the clinical routine and consult with doctors about how such a medical recognition system might fit into the clinical routine.

## 6. Conclusions

The focus of this paper is to demonstrate how Ensemble Learning can be used to perform important and sensitive tasks such as diagnosing COVID-19. We proposed three Ensemble Learning-Based models for COVID-19 detection from chest CT images. Each Ensemble Learning-based model was a combination of pre-trained VGG19, ResNet50, and DenseNet201 networks. We began by preparing the two datasets to be used. We fine-tuned the pre-trained networks by unfreezing a part of each model. We combined the modified models through Stacking and WAE techniques. We used accuracy, precision, recall, and F1-score to compare performance results. We found very encouraging results, especially with the WAE method, which performed the best on the two publicly available chest CT-scan datasets. Consequently, Ensemble Learning, especially the WAE method, is strongly recommended for developing reliable models for diagnosing COVID-19, as well as for a variety of further applications in medicine.

A number of future works are highlighted by the authors. Firstly, the use of chest X-rays datasets to determine whether the ensemble models can be more successful with chest X-ray datasets than with chest CT datasets. Secondly, the use of other Ensemble methods to uncover new findings. Thirdly, the use of some pre-processing techniques to improve the visibility of chest CT images such as gain gradient filter, integrated means filter, etc. Lastly, testing the proposed models in clinical practice and consulting with doctors about their thoughts on these models.

## Figures and Tables

**Table 1:** A summary of the most recent COVID-19 detection methods.

Technique	Modality	Database	Data Augmentation	Transfer Learning	Ensemble Learning	Performance Evaluation
3D segmentation model + location-attention classification model [21]	CT	618 images divided into three classes: COVID-19, viral pneumonia, and healthy people	×	×	×	The overall accuracy obtained is 86.7%
Multi-task learning + Self-supervised learning [22]	CT	COVID-CT dataset	✓	✓	×	An accuracy, AUC, and F1-score of 89%, 98%, and 90%, respectively, is achieved
COVID-Net network [23]	X-Ray	COVIDx dataset: 13,975 CXR images divided into four classes: Normal, bacterial pneumonia, viral pneumonia, and COVID-19	×	×	×	An accuracy of 93% is gained
ResNet50 [24]	X-Ray	COVIDx dataset	✓	✓	×	Attained an accuracy of 96%
Self-supervised Transfer Learning [25]	CT	COVID-CT dataset	✓	✓	×	An AUC and F1-score of 94% and 85%, respectively, is reported
Conditional Generative Adversarial Nets (CGAN) [28]	CT	COVID-CT dataset	✓	✓	×	An accuracy of 76.38% is obtained with AlexNet, 78.89% accuracy with VGG16, VGG19 reaches 73.87%, GoogleNet obtains 77.39%, and ResNet50 gives 82.91%% accuracy
Light CNN based on SqueezeNet [29]	CT	COVID-CT dataset and the Italian dataset	✓	✓	×	83.00% of accuracy, 81.73% of precision, 85.00% of sensitivity, 83% of F1-score, and 81.00% of specificity
2D segmentation model based on U-Net architecture [30]	CT	5212 CT images divided into two classes: COVID-19 and normal	×	✓	×	Obtained a specificity of 88% and a sensitivity of 96%
Joint learning strategy [32]	CT	SARS-CoV-2 CT-scan dataset and COVID-CT dataset	✓	×	×	Achieved 91% accuracy on [42] and 79% accuracy on [22]
Hybrid feature selection [33]	CT	COVID-CT dataset	×	×	×	An accuracy, recall, precision, and F1-score of 96%, 74%, 75%, and 75%, respectively, is gained
Different state-of-the-art pre-trained CNNs [38]	X-Ray	3886 CXR scans divided into three classes: COVID-19, viral pneumonia, and normal	×	✓	×	The most accurate pretrained CNN was VGG16 with 98.29% accuracy
ResNet50V2 network + Modified feature selection pyramid network [39]	CT	63,849 CT scans divided into two classes: COVID-19 and normal	✓	✓	×	Showed 98.49% overall accuracy
ResNet101 [40]	X-Ray	Chest X-ray14 dataset	×	✓	×	Attained an accuracy of 71%, an AUC of 82%, a specificity of 71%, and a recall of 77%
Stacked ensemble [41]	CT	COVID-CTset, SARS-CoV-2 CT-scan dataset, and COVID-CT dataset	✓	✓	✓	Achieved 99% accuracy on [41], 94% accuracy on [42], and 85% accuracy on [22]

CT: Computed Tomography; AUC: Area Under Curve; X-Ray: X-radiation; CGAN: Conditional Generative Adversarial Nets; VGG: Visual Geometry Group; CNN: Convolutional Neural Network; SARS-CoV-2: Severe Acute Respiratory Syndrome Coronavirus; ResNet50: Residual Network-50; AlexNet: Alex Network; GoogleNet: Google Network.

**Table 2:** Distribution of COVID-19 (+) and COVID-19 (−) CT images with respect to their collected sources.

Dataset	Split	COVID-19 (+)	COVID-19 (−)	Total
SARS-CoV-2 CT-scan dataset [42]	Train	1002	984	1986
	Validation	250	246	496
COVID-CT dataset [22]	Train	280	318	598
	Validation	69	79	148

**Table 3:** The parameters selected for Random Forest Classifier and Extra Trees Classifier.

Parameters	Random Forest Classifier	Extra Trees Classifier
n_estimators	200	200
max_depth	15	10
n_jobs	20	20
min_samples_split	30	20

**Table 4:** The hyper-parameters that were used for all Base-Learners.

Network	All Base-Learners Used in This Paper
The number of nodes used in dense layers.	1024
Drop-out rate	0.5
Learning rate	5 × 10^−5^
Mini-batch size	32
Optimizer	Adam
Epochs	50

**Table 5:** Description of the Runtime, Time by epoch, Total parameters, and Best-epoch of the Base-Learners for SARS-CoV-2 CT-scan dataset [42].

Base-Learners	Runtime	Time/Epoch	Total Parameters	Best Epoch
VGG19	1 min	6 s	23,174,210	10/50
ResNet50	3 min 35 s	6 s	43,514,754	31/50
DenseNet201	3 min	7 s	27,238,978	26/50

**Table 6:** Description of the Runtime, Time by epoch, Total parameters, and Best-epoch of the Base-Learners for the COVID-CT dataset [22].

Base-Learners	Runtime	Time/Epoch	Total Parameters	Best-Epoch
VGG19	7 min 16 s	14 s	23,174,210	29/50
ResNet50	11 min 46 s	13 s	43,514,754	50/50
DenseNet201	13 min 14 s	16 s	27,238,978	47/50

**Table 7:** Comparison among the proposed ensemble methods and the Base-Learners on the SARS-CoV-2 CT-scan dataset [42].

Models		Accuracy	Recall	Precision	F1-Score
Base-Learners	VGG19	97.38	96.9	98.04	97.47
	ResNet50	92.96	93.8	92.72	93.26
	DenseNet201	95.57	98.45	93.38	95.85
	Average	95.3	96.38	94.71	95.52
Ensemble methods	2-Levels Stacking	97.59	97.67	97.65	97.08
	3-Levels Stacking	97.59	97.29	98.82	97.67
	WAE	98.59	99.22	98.82	98.65

**Table 8:** Comparison among the proposed ensemble methods and the Base-Learners on the COVID-CT dataset [22].

Models		Accuracy	Recall	Precision	F1-Score
Base-Learners	VGG19	94.13	94.95	93.73	94.34
	ResNet50	79.38	88.32	86.75	78.68
	DenseNet201	91.45	91.37	91.97	91.67
	Average	88.32	86.75	89.92	88.23
Ensemble methods	2-Levels Stacking	93.96	94.79	93.44	94.17
	3-Levels Stacking	94.05	95.6	93.46	94.45
	WAE	95.05	95.28	95.37	94.93

**Table 9:** Comparing the proposed WAE method with methods proposed in previous studies on the SARS-CoV-2 CT-scan dataset [42].

SARS-CoV-2 Ct-Scan Dataset	Accuracy	Recall	Precision	f1-Score
Wang et al. [32]	91	86	96	91
Ebenezer et al. [41]	94	98	90	94
**Proposed WAE Method**	**98.59**	**99.22**	**98.82**	**98.65**

**Table 10:** Comparing the proposed WAE method with methods proposed in previous studies on the COVID-CT dataset [22].

COVID-CT Dataset	Accuracy	Recall	Precision	f1-Score
He et al. [25]	86	–	–	85
Loey et al. [28]	83	78	85	81
Polisinelli et al. [29]	83	85	82	83
Wang et al. [32]	79	80	78	79
Shaban et al. [33]	**96**	74	75	75
Ebenezer et al. [41]	85	95	78	86
**Proposed WAE Method**	95.05	**95.28**	**95.37**	**94.93**

## Data Availability

Not applicable.
